# S. J. E. Wishes the Reader's Opinion on an Uncommon Disease in the Head

**Published:** 1817-01

**Authors:** 


*For the London Medical and Physical Journal.*

S. J. E. *wishes the Reader's opinion on an uncommon Disease in the Head.*

THE following statement of a severe chronic disorder, I beg leave to intrude on your valuable pages, with a hope of receiving instructions how to proceed; trusting, that either the editors, or some of their correspondents, will remit their decision on the cause and the most probable method of cure, through the medium of your Journal.

A married lady, fifty-five years of age, who has borne five children, rather of an irritable disposition, delicate habit, and who has been for the last twenty years continually employed and fatigued with the cares and misfortunes of her family, was, in the month of July 1811, exposed in a cold room after having undergone considerable exertion, which brought on an attack of ophthalmia membranarum, and a catarrh. While her eyes were in this state, she persisted in finishing some needle-work, and constantly used her eyes in this occupation for several days without making use of any antiphlogistic remedy. In the course of a week, she was tacked with an obtuse, and afterwards acute, pain in the head, crossing in a right line from one ear over the ossa parietalia and os frontis to the other; sometimes extending over the whole head, attended with a throbbing which she generally compares to the noise heard when two men are at work on a blacksmith's anvil. Sometimes she imagines that on one side of her head there is violent dashing rain, and, on the other side, an immense hurricane of wind. The pain is always worse after sleep, particularly if she wakes suddenly, which (being very easily roused from slumber) frequently happens to her. After having passed a restless night, she is alarmed in the morning by the dashing in her ears; her pulse, from being commonly slow, is then accelerated; she is thrown into a hot paroxysm ending in perspiration, and generally passes five or six hours in great agony. Any particular exertion, as mental or bodily exercise, talking, reading, writing, &c. augments the disease. She feels herself most free from pain when her mind and body are moderately employed in her domestic concerns, and sometimes she has passed whole days in this manner with very little return of the the pain, or when her bowels have been gently evacuated by some saline cathartic. The disease has not materially altered the whole five years.

It may be necessary to mention, that she is very susceptible of ophthalmia tarsi, and has had several attacks lately. Cessation of the menses has taken place naturally. She has had two or three attacks of peripneumonia, which have all terminated favourably. She is sometimes attacked with a severe, dry, sonorous, cough. Both topical and local blood-letting have been employed without much alleviation; but, once or twice, a slight hæmorrhage from the nose has, for a few days, nearly removed the symptoms. Alterative doses of the hydrargyrus, followed by purgatives, have been administered; blisters have been applied to the temples and nape of the neck, and the antiphlogistic plan has been uselessly adopted. At present, her constitution is much debilitated, and the symptoms continue.

Does this disease arise from a determination of blood to the head? or, is it dependant on some organic affection?

*December* 1816.

---

*For the London Medical and Physical Journal.*

